# Long Term Outcomes of Laparoscopic and Open Modified Lich-Gregoir Reimplantation in Adults: A multicentric comparative study

**DOI:** 10.12669/pjms.334.12661

**Published:** 2017

**Authors:** Arda Atar, Mithat Eksi, Ahmet Faysal Güler, Murat Tuncer, Fatih Akkas, Volkan Tugcu

**Affiliations:** 1 Arda Atar, Bakirkoy Dr. Sadi Konuk Training and Research Hospital, Department of Urology, Istanbul, Turkey; 2 Mithat Eksi, Bakirkoy Dr. Sadi Konuk Training and Research Hospital, Department of Urology, Istanbul, Turkey; 3 Ahmet Faysal Güler, Okmeydani Training and Research Hospital, Department of Urology, Istanbul, Turkey; 4 Murat Tuncer, Kartal Dr. Lutfi Kirdar Training and Research Hospital, Deparment of Urology, Istanbul, Turkey; 5 Fatih Akkas, Bakirkoy Dr. Sadi Konuk Training and Research Hospital, Department of Urology, Istanbul, Turkey; 6 Volkan Tugcu, Bakirkoy Dr. Sadi Konuk Training and Research Hospital, Department of Urology, Istanbul, Turkey

**Keywords:** Laparoscopic, Open, Reimplantation, Ureteric reconstruction

## Abstract

**Background & Objective:**

Obstructive ureteral pathologies in adult patients are most commonly due to ureteral strictures and secondary to surgical interventions. In this study, we aimed to compare open and laparoscopic modified Lich-Gregoir ureteral reimplantation with regards to outcomes in benign ureteral pathologies in adult patients.

**Methods:**

Between December 2008 and December 2014, 32 open cases and 29 laparoscopic cases were performed as per the data retrieved from surgical databases. All laparoscopic procedures were performed in Bakirkoy Dr. Sadi Konuk Training and Research Hospital(BEAH) and all open ureteral reimplantation procedures in Kartal Dr Lutfi Kirdar Training and Research Hospital(KEAH) and Okmeydani Training and Research Hospital(OEAH).

**Results:**

The mean operation time was significantly lower in the group of patients operated with open group (142.5 minutes versus 188.9 minutes; P< 0.0001). The mean duration of follow-up was longer in the laparoscopy group (31 versus 28 months; p< 0.0001). The mean amount of operation associated blood loss was significantly lower in patients operated laparoscopically (93.7 mL versus 214 mL; P< 0.0001). The mean VAS score obtained six hours after surgery was 6.6 ± 0.8 in open group, and 5.8 ± 0.7 in laparoscopic group (p=0.0004). The mean VAS scores measured at post-operative day 1 was 4.5 ± 0.7 in open group and 3.7 ± 0.9 in laparoscopy group. Time required to achieve the pre-operative capability of daily activities was significantly longer in open group (15 ± 1.4 days vs 11 ± 1.4 days; p< 0.0001).

**Conclusion:**

Despite open techniques provide shorter operation time and laparoscopic techniques require long learning curve, we think that laparoscopic techniques are superior to open ones since that they provide a better post-operative comfort and are better tolerated in terms of complications.

## INTRODUCTION

In adult patients, obstructive ureteral pathologies are most commonly associated with ureteral stenosis secondary to surgical interventions. Nonsurgical causes of ureteral strictures are namely infections (tuberculosis), ureteral valve, gunshot injury and inflammatory diseases (endometriosis, pelvic inflammatory disease). If the stricture is short and the endoscopic treatment attempt is unsuccessful, the lower ureteric defects can be managed with ureteroureterostomy and ureteroneocystostomy. For longer defects, complex techniques such as vesico-psoas hitch, Boari-flap, ileal ureteral substitution or even autotransplantation have been successfully used.

Although open technique is still the gold standard, with developing technology, laparoscopic and robotic technics are being decribed and increasingly used by authors. With laparoscopic techniques, the amount of bleeding, the length of stay in the hospital, the postoperative pain and the length of stay in the hospital were reduced.^1^-^3^

In this study, we aimed to compare open (OG) and laparoscopic (LG) modified Lich-Gregoir ureteral reimplantation with regards to outcomes in benign ureteral pathologies in adult patients.

## METHODS

After approval of the Hospital Ethics Committee, a retrospective analysis of the medical records of the patients was performed in three specialized urology centers (Bakirkoy Dr. Sadi Konuk Training and Research Hospital (BEAH), Okmeydani Training and Research Hospital (OEAH) and Kartal Dr. Lutfi Kirdar Training and Research Hospital (KEAH), Istanbul, Turkey).

A retrospective survey of surgical databases of three instutions for ureteroneocystostomy revealed 32 open cases and 29 laparoscopic cases from December 2008 to December 2014. All laparoscopic procedures were performed in BEAH and all open ureteral reimplantation procedures in KEAH and OEAH. Patients older than 21 years, with a unilateral distal ureteral defect less than four cm and a follow-up time longer than 24 months were included in this study. Patients having primary vesicoureteral reflux, distal ureteral carcinoma and undergoing immediate reconstruction for ureteral injury were excluded. The demographics of the patients and the indications for reimplantation are summarized in Table-I.

**Table-I:** Demographic characteristics of the patients.

	Open	Laparoscopic	p value
Patients	32	29	
Age (yr)			
Mean ± SD	49.8 ± 5.6	51.9 ± 6.6	0.046
Range	(34-63)	(38-62)	
Left Side (n)	17	16	0.93
Men (n)	9	6	0.96
Body Mass Index (kg/m^2^)			
Mean ± SD	26.9 ± 1.7	27 ± 2	0.742
Range	(22-30)	(22-31)	
Preoperative serum creatinine (mg/dL)			
Mean ± SD	0.9 ± 0.2	0.8 ± 0.1	0.641
Range	(0.7-1.5)	(0.6-1.5)	
Stricture Length (cm)			
Mean ± SD	2.17± 0.9	2.12 ± 0.9	0.823
Range	(1-4)	(1-4)	
Indications for ureteral reimplantations			
Ureteral valve	1	0	
Urinary tuberculosis	2	1	
After open ureterolithotomy	2	0	
Secondary to multiple endoscopic stone therapy	4	3	
Inflamatory diseases*	2	2	
After open ureteral reimplantation	3	4	
Previous gynecological surgery			
Hysterectomy	8	4	
Cesarian section	2	2	
Ovarian tumor	3	1	
Cervix cancer (Wertheim)	1	2	
Ureterovaginal Fistula	2	6	
Previous abdominal surgery			
Rectal carcinoma	2	4	

* Endometriosis, chronic pelvic inflammatory disease.

In order to assess the length and location of the stricture, intravenous, antegrade or retrograde urography and dynamic synthgrapy were used. An ureteral stent or percutaneous nephrostomy tube was placed before evaluating the renal functions. Operation time, blood loss, complications, VAS pain scores on the day of operation and postoperative day one, duration of hospital stay, time required to achieve the pre-operative capability of daily activities were recorded for each patient. VAS scale was used to evaluate patient-assessed severity of pain. While interpretating VAS scores, 0 represents minimum (no) pain and 10 maximum (the worst possible) pain. Serum urine and creatinine levels, ultrasonography, intravenous urography and dynamic synthgrapy were performed during the follow up and if indicated, retrograde urography was also performed.

SPSS version 22.0 (IBM SPSS, USA) software was used for statistical analysis. The results were presented as basic parameters of descriptive statistics. The distribution of variables was evaluated using Kolmogorov- Smirnov test. Continuous data were analyzed using independent samples t-test or Mann-Whitney U test.

### Surgical Technique

The surgical technique was performed as previously described. The laparoscopic extravesical modified Lich Gregoir ureteral reimplantation technique reproduced open surgical steps.^4^-^6^ Under general anesthesia, a 14F Foley catheher was placed in the bladder and patients were placed in a supine position with Trendelenburg maneuver. Pneumoperitoneum was created with a Veress needle and the 12-mm camera port was placed infraumblically at the midline. As for the surgical instruments, two trochars were placed on the midclavicular line for both left and right and for additional assistance, a 5 mm trochar was placed on the anterior axillary line above the iliac crest.

The colon was mobilized through a dissection from the Toldt line. The ureter was defined at the iliac vein level and dissection was continued through ureterovesical junction until the area of stenosis was identified. The strictured ureteral segment was clipped with the help of a hemoclip. The proximal ureteral segment of strictured segment was spatulated. The bladder was filled with serum saline and the affected ureter was dissected up to the bladder. Detrusor incisions were made in accordance with the ureteral diameter. The submucosal tunnel was created and the mucosa was detached from the distal end of the tunnel. The spatulated ureter and mucosa were then sutured and a D-J catheter was placed before the final suture. The detrusor was closed to support the submucosal tunnel and the psoas hitch was applied.

The drain was withdrawn if the drainage level fell below 50cc. The urethral catheter was removed on post operative day seven if there was no sign of urinary leakage on the cystography. The D-J catheter was removed 6-8 weeks later. Complications were reported according to the Clavien-Dindo grading system for the classification of surgical complications.^7^

## RESULTS

The baseline patient demographics including age, sex, preoperative serum creatinine, body mass index and side of involvement were not statistically different between the two groups as shown in Table-I. The mean stricture length was similar between the two groups (p= 0.823). The most common etiology of stricture was iatrogenic in both groups. The outcomes of the open and laparoscopic ureteral reimplantation are reported in Table-II. The mean operation time was significantly lower in the OG (142.5 min versus 188.9 min; P< 0.0001). The mean duration of follow-up was longer in the OG (31 versus 28 months; p< 0.0001). The mean operative blood loss was significantly lower in the LG (93.7 mL versus 214 mL; P< 0.0001). At six hours postoperatively, the mean VAS score was 6.6 ± 0.8 in OG, and 5.8 ± 0.7 in LG (p=0.0004). The mean VAS scores at post operative day 1 was 4.5 ± 0.7 in OG and 3.7 ± 0.9 in LG. The VAS scores were significantly higher in OG. The median duration of hospital stay was significantly shorter in the LG (2.9 ± 0.8 days versus 5.5 ± 1 days; p< 0.0001). Time required to achieve the pre-operative capability of daily activities was significantly longer in OG (15 ± 1.4 days vs 11 ± 1.4 days; p< 0.0001). Laparoscopic ureteral reconstruction was technically feasible in all patients and did not neccessitate an open conversion in any.

**Table-II:** Outcomes of ureteral reimplantation.

*Variable*	*Open*	*Laparoscopic*	*p value*
Patients (n)	32	29	
***Operation time (min)***			
Mean ± SD	142.5 ± 21	188.9 ± 18.3	< 0.0001
Range	(110-220)	(160-220)	
***Blood Loss (ml)***			
Mean ± SD	214 ± 35.7	93.7 ± 19.5	< 0.0001
Range	(140-250)	(70-130)	
***VAS Day 0 (point)***			
Mean ± SD	6.6 ± 0.8	5.8 ± 0.7	p= 0.0004
Range	(5-8)	(5-8)	
***VAS Day 1 (point)***			
Mean ± SD	4.5 ± 0.7	3.7 ± 0.9	p= 0.0004
Range	(3-6)	(2-6)	
***Length of hospitalization (day)***			
Mean ± SD	5.5 ± 1.0	2.9 ± 0.8	< 0.0001
Range	(4-7)	(2-5)	
***Return to Normal Activities (day)***			
Mean ± SD	15 ± 1.4	11 ± 1.4	< 0.0001
Range	(11-17)	(9-15)	
***Duration of follow-up (month)***			
Mean ± SD	31 ± 1.6	28 ± 2.2	
Range	(28-33)	(25-32)	< 0.0001

VAS: Visual Analogue Scale

Postoperative complications are summarized in Table-III. Two patients in the OG manifested with prolonged ileus and treated conservatively. Postoperative fever due to urinary tract infection was evident in one patient in OG and treated with antibiotherapy. Urinary leakage was present in one patient in LG and therefore the time of bladder decompression was prolonged with urethral catheterisation.

**Table-III:** Complications according to the Clavien grading system.

	Open	Laparoscopic
***Grade 1***		
Ileus	2	-
***Grade 2***		
Fever	1	-
Urinary Leak	-	1
***Grade 3b***		
Restricture	1	1

One patient who underwent laparoscopic ureteroneocystostomy due to chronic pelvic inflammatory disease and one patient who underwent open ureteroneocystostomy due to urinary tuberculosis were reported to have recurrent stenosis during the follow up. They were treated with endoscopic balloon dilation and double-J stent application. With the exception of these two patients, intravenous urographic imaging showed normal or mild residual hydronephrosis, and kidney function was normal during dynamic synthgrapy studies for each patient. We have not reported any reflux or stenosis.

## DISCUSSION

Distal ureteral strictures can be seen as a result of congenital pathologies, chronic inflammation, malignancy, trauma, radiotherapy or iatrogenic injuries^1^. Patients with a history of multipl endourologic interventions secondary to the stone diseases may have distal ureteral stricture by the contribution of anatomical narrowness^8^. The rates of ureteral stricture in patients who previously underwent ureterorenoscopy and laser lithotripsy is reported in 0-4% by different series.^9^ In all surgical procedures, the rate of iatrogenic ureter injury is reported in 0.01% to 2.5%.^10^,^11^ More than half of these cases are secondary to gynecological interventions.^12^ In our study; 31 patients (50.8%) had gynecologic, 16 (26.2%) had urologic interventions, while 6 (9.8%) had a history of abdominal surgery.

Generally short stenosis in the distal ureter is treated with ureteroureterostomy and ureteroneocystostomy, in complicated forms of stenosis there is a need of advanced techniques, namely vesicopsoas hitch and Boari flap.^13^ We performed vesicopsoas hitch for each patient in order to obtain tension-free anastomosis.

Successful results of laparoscopic and robotic technicques in urological reconstructive surgery were reported in previous series.^4^,^14^-^16^ However, since these techniques require a longer time to be learned and the operation time is prolonged, open surgical approach is still the gold standard of care.^17^

Laparoscopic surgery provides lower amounts of blood loss, less postoperative pain, shorter hospital stay and shorter recovery periods compared to open surgery.^4^,^18^ The data obtained from our study support these findings. In the laparoscopy group, although the operation time was significantly longer, there were pronounced advantages with a less amount of bleeding, lower postoperative VAS scores, shorter hospital stay and a rapid recovery.

Tension-free, well vascularized and water-tight anastomosis is essential to avoid ischemic complications, restriction and to provide better outcomes.^19^,^20^ Improved dissection and anastomosis can be achieved in laparoscopic and robotic surgery with the clarification and enlargement of visual field with the help of bleeding-free working area obtained with the buffering effect of pneumoperitoneum.^4^,^19^

Rate of complications reported for open and laparoscopic ureteroneocystostomy in previous series range from 8% to 20%.^8^,^15^,^19^ In our study, four patients (12.5%) in OG and two patients (6.8%) in LG had complications. The rates of complications on the other hand, were not significantly different between the two groups. (p= 0.46).

One of the most common complications seen after reconstructive ureteric surgery is recurrent stenosis of 5%.^21^ In our study, restricture was reported in two patients (3.2%). Both of these patients had a history of ongoing chronic inflammatory process. While this evidence suggests chronic/systemic inflammation as a risk factor for restricture, further investigation is required to confirm our findings.

The main limitation of our study was the retrospective design. Moreover, differences in the peroperative and postoperative measurements due to multicentric nature might be another possible problem.

## CONCLUSION

Management of short distal ureteral strictures with open and laparoscopic ureteroneocystostomy procedures are successful and effective. Although the duration of operation is shorter with open techniques and laparoscopic interventions require longer time to reach proficiency, we suggest that laparoscopic techniques are superior to open techniques when well pronounced postoperative comfort and lower rates of complications are concerned.

### Authors’ Contributions

**AA, ME, FA** conceived, designed and did statistical analysis & editing of manuscript.

**AA, ME, AFG, MT and FA** did data collection and manuscript writing.

**AFG, MT, VT** performed operations and did review and final approval of manuscript.
